# Using rapid cycle deliberate practice to improve primary and secondary survey in pediatric trauma

**DOI:** 10.1186/s12909-020-02038-z

**Published:** 2020-04-28

**Authors:** Diana Hou Yan, Mark B Slidell, Alisa McQueen

**Affiliations:** 1 Department of Pediatrics, Section of Emergency Medicine, University of Chicago Medicine, 5841 S Maryland Ave, Mailcode 0810, Chicago, IL 60637 USA; 2 Department of Surgery, University of Chicago Medicine, 5841 S Maryland Ave, Chicago, IL USA

**Keywords:** Medical curriculum, Trauma, Education, Surgery simulation

## Abstract

**Background:**

Optimal performance of the primary and secondary survey is the foundation of Advance Trauma Life Support care. Despite its importance, not all primary surveys completed at level 1 pediatric trauma centers are performed according to established guidelines (Gala et al., Pediatr Emerg Care 32:756–762, 2016, Carter et al., Resuscitation 84:66–71, 2013). We hypothesize that rapid cycle deliberate practice (RCDP) will improve surgical residents’ confidence in performing the primary and secondary survey.

**Methods:**

We developed a curriculum to teach surgical interns the principles of performing the primary and secondary survey using RCDP. Surveys distributed after each session assessed the impact of the curriculum on learner confidence and perception that this curriculum would benefit patient care. Questions were scored on a 5-point Likert scale. Sixteen surgical interns participated during intern orientation and 100% of the participants completed the post curriculum survey.

**Results:**

Thirteen (81%) of participants agreed or strongly agreed that the simulation would impact future performance in the pediatric trauma bay. The curriculum also significantly improved the confidence of our learners to perform trauma surveys (*p* < 0.001).

**Conclusion:**

This curriculum improves the confidence of junior surgical residents in learning the primary and secondary survey. Most learners enjoyed the session and felt that the curriculum would positively impact their performance.

## Background

Trauma is a leading cause of death in the United States with almost 200,000 people dying due to injury annually [1]. Advance Trauma Life Support (ATLS), developed in the 1970s, is the basis of trauma care and has systemically improved its care [2, 3]. The foundation of ATLS care is the primary and secondary survey. These two surveys identify the traumatic injuries sustained and determine the resuscitation needs while the patient is in the trauma bay [4]. A recent study showed that only 22% of primary surveys of level 1 pediatric traumas at a pediatric trauma center were performed according to established guidelines and omitted important elements [5]. Additionally, Carter et al. showed that only 13% of resuscitations completed all primary and secondary tasks [6]. These studies demonstrate a gap in these early steps of ATLS care. Additionally, we found that only 22.4% of secondary surveys in our own intuition completed all parts of the secondary survey (data not shown). Given this initial needs assessment, we created a simulation-based curriculum focusing on the accurate performance of the primary and secondary survey.

Multiple studies have shown that simulation is an effective tool for teaching trauma care [7]. Learners enjoy the training [8–10] and that they believe they have learned something from the simulation that will affect patient care [10]. Studies have used video or in situ evaluations to show behavior changes in simulated patient scenarios [8, 11, 12]. Rapid cycle deliberate practice (RCDP) is an innovative simulation method that uses coaching to educate learners by allowing them to maximize their time spent in deliberate practice to create accurate muscle memory in a safe environment [13]. It uses direct feedback to adjust how learners are doing specific tasks so that they can have multiple opportunities to “try again.” This allows them to build on their skills and replaces the lengthy debrief at the end of a traditional simulation. This method of simulation has been shown to effectively teach resuscitation to pediatric residents, but has not yet been performed with surgical residents [9, 13–15]. It can be particularly effective for primary and secondary survey training as it is repetitive and algorithmic. There is a particular order in which it is taught to the surgical residents so that elements of the survey are not forgotten and all injuries are identified in a trauma patient.

We hypothesized that a focused simulation teaching curriculum using RCDP could improve the confidence of surgical residents in performing the primary and secondary survey in pediatric trauma.

## Methods

This project was formally determined to be quality improvement, not human subjects research, and was therefore not overseen by the Institutional Review Board, per institutional policy.

The trauma survey curriculum began with an instructional video, created by the authors, describing how to perform the primary and secondary survey in a pediatric patient. Participants then applied the primary and secondary survey skills in small groups using RCDP in simulated pediatric cases. All sessions were conducted during intern orientation (June 2018). Sixteen learners participated during intern orientation in 5 groups with the longest session lasting 1 h. The same instructor conducted all 5 teaching sessions.

Each RCDP session warns learners that there will be multiple interruptions during the simulation session, and includes an explanation of the functionality of the mannequin.

Once introductions are complete, one learner begins the primary survey. When one major or two to three minor errors occur, the instructor stops the learner and corrects them. The learner restarts a few steps before the initial error in order to build strong and accurate muscle memory. The first case is always a completely normal exam so that learners can build on the group’s knowledge and advance in difficulty case after case. The cases developed for this curriculum (Additional file 1) are:

- Case 1: Pedestrian versus Automobile – 5 year old male who was hit by a car going at 5 miles per hour. Primary and secondary survey are normal.
- Case 2: All-Terrain Vehicle (ATV) Accident – 12 year old male who’s right leg was ran over by ATV after falling out of ATV. Primary survey is normal, and secondary survey is significant for femur fracture of right leg and left tibia/fibula fracture.
- Case 3: High Speed Motor Vehicle Accident – 4 year old male who was in a motor vehicle collision with major damage to the car. Primary survey shows no breath sounds on the right side with deviated trachea to the right. Secondary survey is significant for tenderness and bruising over the chest.
- Case 4: House Fire – 1 year old female who sustained burns after being in a house fire. Primary survey shows whimpering child and secondary survey shows soot in mouth and burns on chest and abdomen.
- Case 5: Bicycle versus Car – 15 year old male who was in a bicycle versus car with the car going at 30 miles per hour. Primary survey shows a Glasgow Coma Scale of 8 indicating intracranial injury and secondary survey is significant for contusion on head and abrasions to extremities.
- Case 6: Shooting – 12 year old male who sustained a gunshot wound (GSW) to the right chest. Primary survey is significant for right sided hemothorax (deviated trachea to the left and no breath sounds on the right) and secondary survey is significant for crepitus of the right chest and GSW holes.

In this offering of the curriculum, while the first case was always used, the other cases were randomly distributed among the separate offerings.

**Fig. 1:**
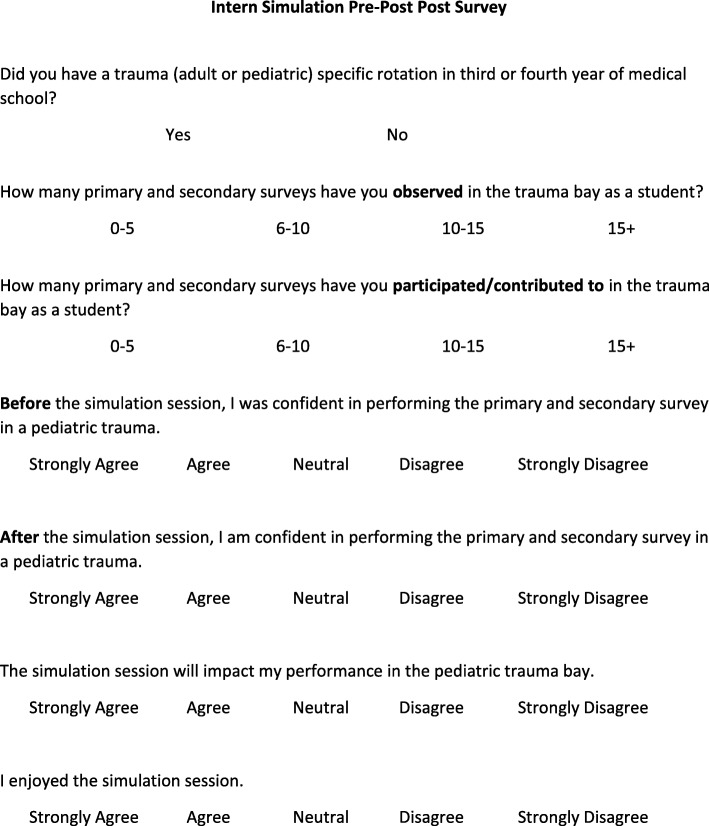
Intern Simulation Pre-Post Survey. This survey is scored on a 5-point Likert scale and was distributed after each session to assess the impact of the curriculum on learner confidence and perception

At the end of each session, learners were surveyed (Fig. 1) to assess their confidence and whether they believed the simulation would affect their care through 4 evaluation questions, each on a 5-point Likert scale (1 = strongly disagree to 5 = strongly agree). Demographic questions assessed baseline trauma experience. Comparison of post- vs. pre-session confidence levels was performed using the Wilcoxon signed-rank test. Assessment of the magnitude of confidence level changes across types of learners was made using the Kruskal-Wallis test. Analyses were performed using Stata 15 (StataCorp., College Station, TX).

## Results

**Fig. 2:**
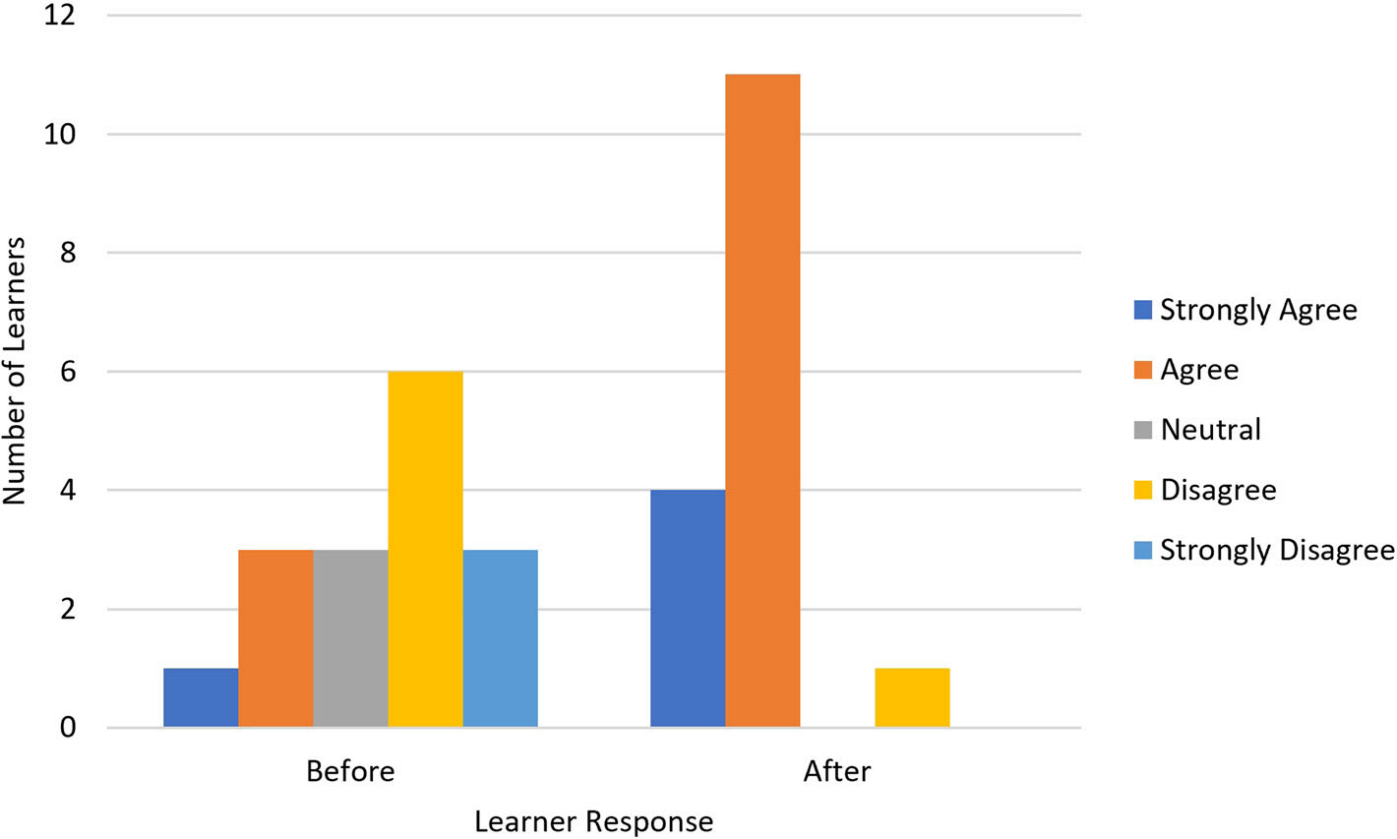
Aggregate data (*n* = 16) of learner responses to their confidence before and after the simulation session. *p*-value < 0.001. This is aggregate data of learner responses to the confidence question on the survey distributed at the end of each simulation session. The confidence question asks learners what their confidence is to perform a primary and secondary survey before and after the simulation session

**Fig. 3:**
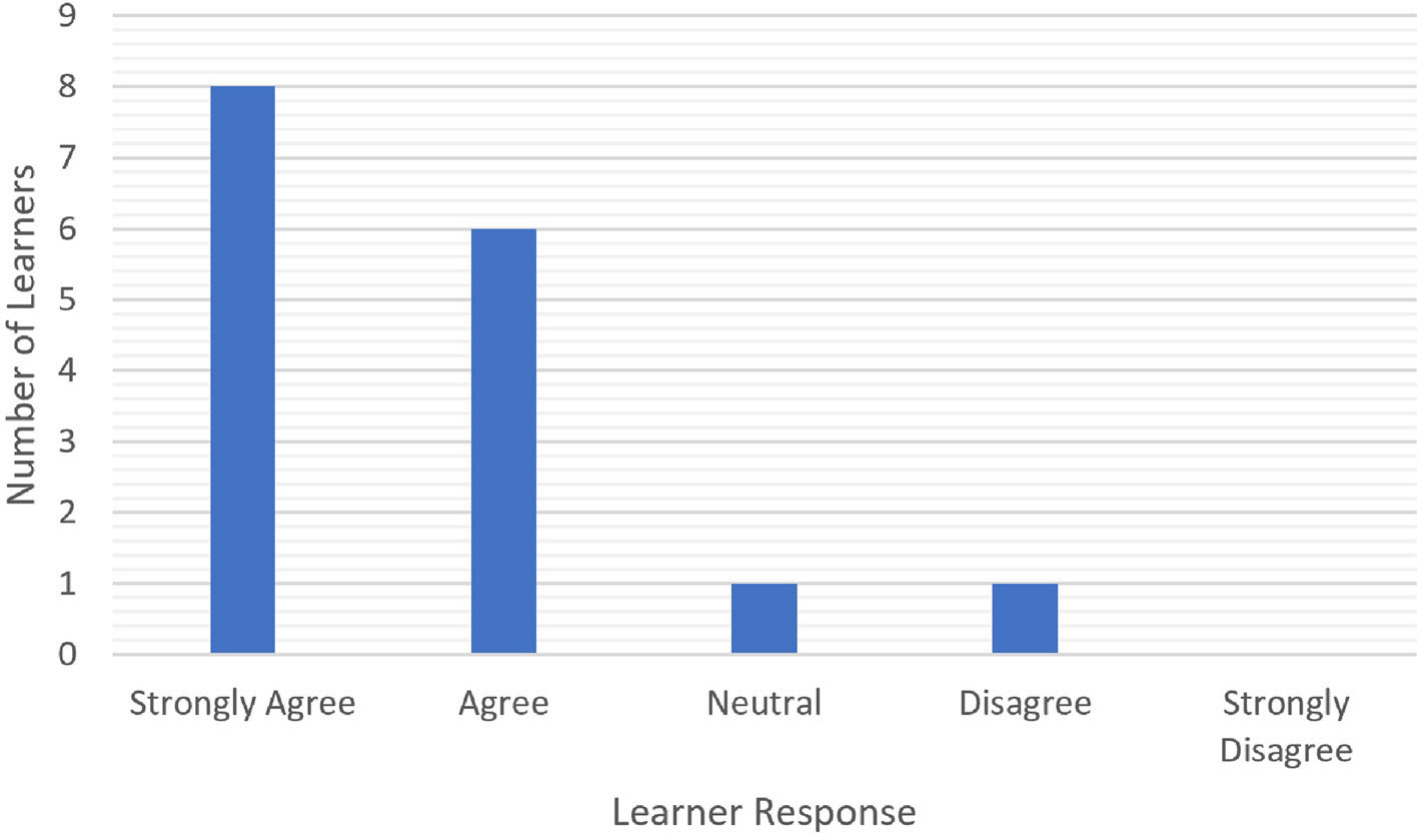
Aggregate data (*n* = 16) of learner responses to their enjoyment of simulation session. This is aggregate data of learner responses to their enjoyment of the simulation session on the pre- vs. post-session survey. The enjoyment question is to access if the learners liked the simulation session

**Fig. 4:**
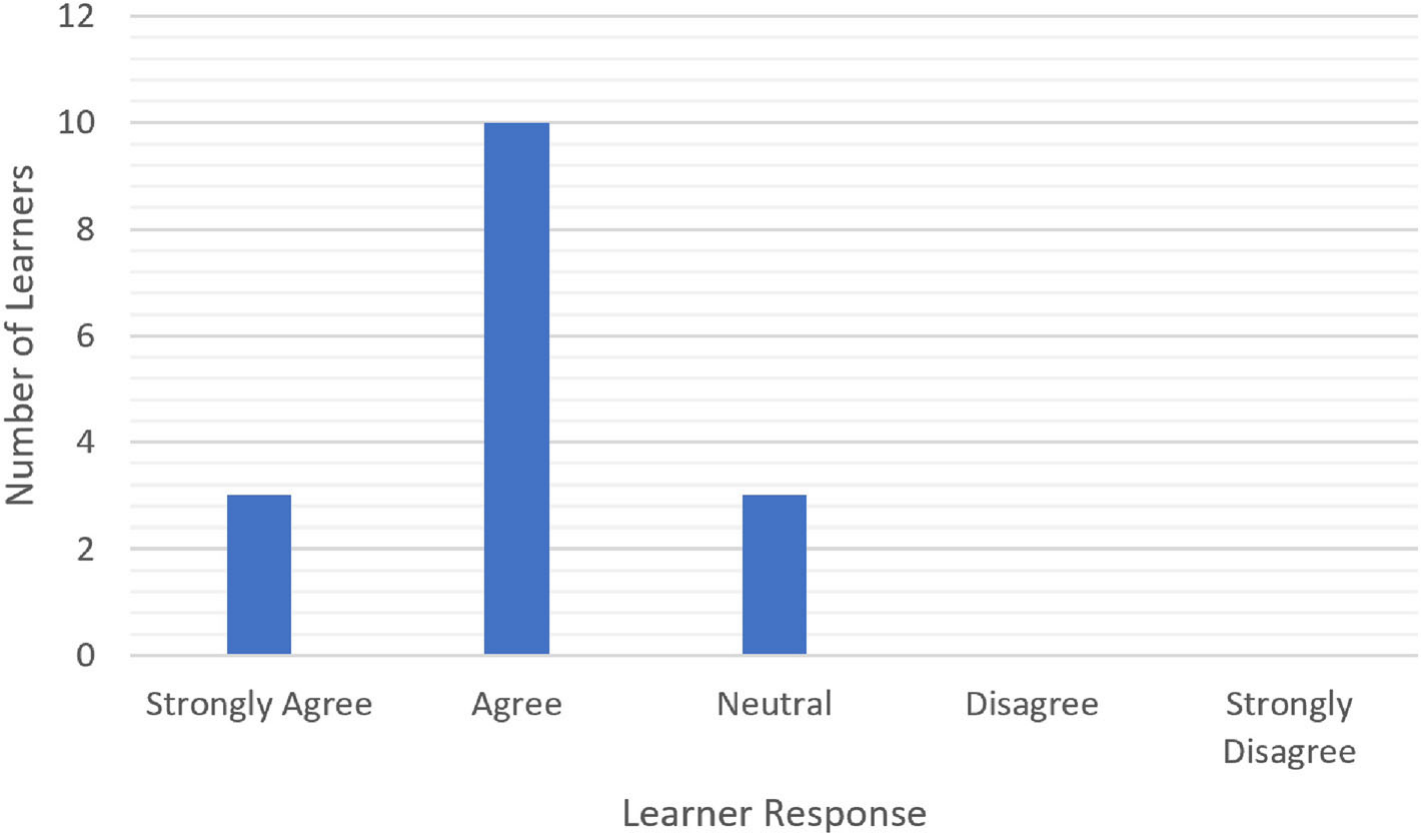
Aggregate data (n = 16) of learner responses to if the simulation session will impact performance in pediatric trauma bay. This is aggregate data of learner responses to whether or not the residents felt that the simulation session could positively affect their performance in the pediatric trauma bay. This question is to assess translation of what is learned in simulation to clinical practice

There was a significant improvement in learner confidence after the curriculum (*p* < 0.001, Fig. 2). 9 of 16 learners have a negative response to confidence prior to the simulation session and 14 of 16 learners gave higher confidence ratings compared to prior to the session (Table 1). Interestingly, magnitude of this change was greater for the otolaryngology, urology and plastic surgery residents than for the general surgery residents (*p* = 0.009). 14 of 16 learners enjoyed the simulation session and gave a response of strongly agree or agree (Fig. 3). Thirteen of 16 learners (81%) felt that the simulation session would impact their performance in the pediatric trauma bay (Fig. 4). Each session ranged from 20 min to a 1 h (Table 2). The total time for all 16 learners was 185 min.

**Table 1:** Individual responses of before and after questions. These are the actual individual responses to the question about learner confidence before and after the RCDP simulation session

Learner Number	Before Simulation Confidence	After Simulation Confidence
1	Disagree	Agree
2	Strongly Disagree	Agree
3	Disagree	Agree
4	Strongly Disagree	Agree
5	Disagree	Strongly Agree
6	Disagree	Agree
7	Disagree	Agree
8	Neutral	Agree
9	Neutral	Agree
10	Agree	Strongly Agree
11	Agree	Strongly Agree
12	Strongly Disagree	Disagree
13	Strongly Agree	Strongly Agree
14	Neutral	Agree
15	Disagree	Agree
16	Agree	Agree

**Table 2:** Time spent for each session with learners. This table describes the number of learners and time spent on each session. The first two sessions were with general surgery interns and the other sessions were with surgical subspecialty interns

Session Number	Number of Learners	Time Spent (minutes)
1	3	30
2	6	60
3	2	30
4	2	20
5	3	45

## Discussion

Our curriculum led to a significant improvement in surgical resident confidence to perform the primary and secondary survey. While examining patient outcomes was beyond the scope of this project, this confidence could translate to improvement in actual performance in pediatric trauma care. Most learners enjoyed the session and felt that the curriculum would positively impact their performance in the trauma bay.

RCDP applies a coaching method to simulation. Because traditional simulation is increasingly used in medical training, residents are often familiar with the model of conducting a full simulation and then debriefing at the end. RCDP breaks this model into pieces. It can be disconcerting to the learner for the instructor to stop and correct them during a simulation. Once learners are aware that this will happen even if they are excelling, it normalizes the situation and prevents learners from building a defense when they are interrupted. It is important to ensure that the learners know that this is a safe place to make mistakes and that errors are expected. The survey results showed that this innovative method can increase the confidence of surgical residents in performing the primary and secondary survey in a pediatric trauma patient. Additionally, with the addition of harder and harder cases, the learners progressed from a novice learner to a master performer.

In addition to improving a surgical resident’s confidence and knowledge, this work also applies RCDP in a new way – to trauma care. While there have been many examples of RCDP in other settings such as Basic Life Support and Pediatric Advance Life Support, little is published about the application of RCDP to pediatric trauma care education [11, 13]. The one other published work involving RCDP in pediatric trauma education described a longitudinal simulation curriculum for pediatric emergency medicine fellows, with one trauma case in a 12 month curriculum [16]. Additionally, RCDP is useful when there are significant time limitations. RCDP took about 10–15 min per learner and most sessions were 20–30 min long (Table 2). Therefore, a one-hour session with 6 learners and 6 cases can be accomplished. This is a major advantage of RCDP as learners are able to experience multiple cases of increasing difficulty and their time is used to build the correct motor skills for application to the care for injured trauma patients.

It was interesting that in an exploratory analysis, there was a smaller improvement in confidence in the general surgery group compared to other groups. This is possibly because this group had just received ATLS certification the week prior to when this curriculum was completed. Despite them having just completed ATLS, there was still a trend toward improvement in their confidence which shows that this curriculum can be used even in this scenario. Additionally, there were general surgery residents who underwent ATLS who still gave a negative response to confidence before the RCDP session.

There are several limitations. Our sample size was small due to the number of available interns in an academic year. This can be improved upon in future work by including subsequent years’ learners. Additionally, self-reported data introduces the possibility of reporting bias.

## Conclusion

This study shows that surgical residents’ confidence can be significantly improved after completing a RCDP-based simulation training on primary and secondary survey in pediatric trauma care. Future directions include assessment of surgical residents’ actual performance in order to assess the degree to which self-confidence translates to clinical skills. Other possibilities are to apply this curriculum to other practitioners such as advance practice registered nurses and pediatric emergency medicine fellows or to do a longitudinal RCDP-based curriculum.

## Supplementary information

**Additional file 1.** Simulation Cases. Description: These are the six cases that were created for this trauma simulation curriculum.

## Data Availability

The datasets used and/or analyzed during the current study available from the corresponding author on reasonable request.

## Abbreviations

ATLS: Advance trauma life support
ATV: All-terrain vehicle
GSW: Gunshot wound
RCDP: Rapid cycle deliberate practice
