# Supplementary material for: Using rapid cycle deliberate practice to improve primary and secondary survey in pediatric trauma
Source: BMC Med Educ. 2020 Apr 28;20:131. doi: 10.1186/s12909-020-02038-z (PMC7189590; doi:10.1186/s12909-020-02038-z)
Supplement: Supplementary file 1 — Additional file 1. Simulation Cases. Description: These are the six cases that were created for this trauma simulation curriculum. [file 12909_2020_2038_MOESM1_ESM.docx]

**Simulation Cases Appendix**

**Case 1**

Title: Pedestrian versus Automobile

Author: Diana Yan

Patient Name: John Doe

Patient Age: 5 year old

Chief Complaint: Peds vs Auto

Objective:

1. Review the steps of a timely and complete primary and secondary survey.

Expected Run Time: 5-7 min

Simulation Location: Pediatric Trauma Bay

Scenario Participants: Surgical Residents – one is doing primary and secondary survey; one is holding airway; rest are observers; foot of bed/leader is facilitating the simulation

Props needed: C collar on mannequin, gown for mannequin that can be “cut” off, stethoscope, warm blankets off to the side

Scenario:

Trauma Pager: Level 2 Trauma: 5 yo male Peds vs Auto. ETA 5 min.

EMS report: This is a 5 year old male was in peds vs car going at 5mph on neighborhood streets. Patient is awake and talking. No LOC at scene. No complaints of pain. 105 HR, 105/Palp, 22, 99%O_2_ Sat.

Starting vitals: T 36.8, HR 107, RR 20, BP 108/70, Pox 98% on room air

Mannequin: Normal breath sounds, bowel sounds, heart sounds (no murmur), eyes open, not talking unless asked questions

Resident will start to perform primary survey (normal exam):

- Airway (<10 sec): ask patient to talk and observe that patient is talking (airway patency), no tracheal deviation
- Breathing (<10 sec): Auscultation in two lung fields, inspection of equal chest rise
- Circulation (<10 sec): Palpation of pulses, note no obvious bleeding
- Disability (<30 sec): Size and responsiveness of bilateral pupils, GCS 15
- Exposure: “cut” clothes off and get warm blankets to prevent hypothermia

If have errors, will stop and reset, review and restart again. Make sure that they have looked at vitals at least once.

Resident will start to perform secondary survey (normal exam): [Resident will ask for different exam findings and facilitator will give normal findings.]

- Head (<10 sec): Palpation/Inspection of scalp, otoscope exam
- Face (<10 sec): Palpation/Inspection of face, oropharyngeal exam
- Neck (<10 sec): Inspection of anterior neck
- Chest wall (<10 sec): Palpation of chest wall, evaluation of tenderness/crepitus
- Abdomen (<10 sec): Palpate abdomen for tenderness, soft vs firm, distension
- Pelvis (<10 sec): Palpation for tenderness (including pubic symphysis) and rocked correctly
- Perineum/Urethra (<10 sec): Visual exam of perineum (including meatus in males) and inspect for blood
- Extremities (<20 sec): Visual exam and palpation for tenderness of all non-deformed aspects of 4 limbs
- Axillae (<10 sec): Inspect bilateral axillae
- Neuro (< 20 sec): Test for strength/sensation of each limb and how extremity is held
- Back (<20 sec): Palpation for stepoffs and tenderness along C/T/L spine, rectal inspection

If have errors, will stop and reset, review and restart again.

At end, final checklist of saying yes or no to completion of tasks in primary and secondary survey.

**Case 2**

Title: ATV Accident

Author: Diana Yan

Patient Name: Jessie Doe

Patient Age: 12 year old

Chief Complaint: ATV Accident

Objectives:

1. Review the steps of a timely and complete primary and secondary survey.
2. Be able to recognize right femur fracture and find new left tib/fib fracture, highlighting importance of complete primary and secondary survey in all patients.

Expected Run Time: 5-7 min

Simulation Location: Pediatric Trauma Bay

Scenario Participants: Surgical Residents – one is doing primary and secondary survey; one is holding airway; rest are observers; foot of bed/leader is facilitating the simulation

Props needed: C collar on mannequin, gown for mannequin that can be “cut” off, stethoscope, warm blankets off to the side, ACE wrap on right leg

Scenario:

Trauma Pager: Transfer from outside ED: 12 yo M with right femur fracture. ETA 7 min.

EMS report: This is a 12 year old male who was in ATV accident. Was wearing helmet. No LOC. Right leg was run over by another ATV when patient fell out of his ATV when going over bump. Transferred from another hospital due to right femur fracture that was found on X-ray. He already got two doses of morphine. Patient is awake and complaining of pain. 115 HR, 125/75, 22, 99%O_2_ Sat.

Starting vitals: T 37.0, HR 122, RR 24, BP 127/80, Pox 100% on room air

Mannequin: Eyes open, crying, normal breath sounds, bowel sounds, heart sounds (no murmur)

Resident will start to perform primary survey (normal exam):

- Airway (<10 sec): patient is crying in pain (airway patency), no tracheal deviation
- Breathing (<10 sec): Auscultation in two lung fields, inspection of equal chest rise
- Circulation (<10 sec): Palpation of pulses (if palpate R femoral, then patient will start crying louder), note no obvious bleeding
- Disability (<30 sec): Size and responsiveness of bilateral pupils, GCS 15
- Exposure: “cut” clothes off and get warm blankets to prevent hypothermia

If have errors, will stop and reset, review and restart again. Make sure that they have looked at vitals at least once.

Resident will start to perform secondary survey (pain in bilateral lower extremities): [Resident will ask for different exam findings and facilitator will give findings. ACE wrap on patient will indicate splint/abnormal finding.]

- Head (<10 sec): Palpation/Inspection of scalp, otoscope exam
- Face (<10 sec): Palpation/Inspection of face, oropharyngeal exam
- Neck (<10 sec): Inspection of anterior neck
- Chest wall (<10 sec): Palpation of chest wall, evaluation of tenderness/crepitus
- Abdomen (<10 sec): Palpate abdomen for tenderness, soft vs firm, distension
- Pelvis (<10 sec): Palpation for tenderness (including pubic symphysis) and rocked correctly
- Perineum/Urethra (<10 sec): Visual exam of perineum (including meatus in males) and inspect for blood
- Extremities (<20 sec): Visual exam (deformed right femur in ACE wrap/splint), cries louder when right leg is touched and asks examiner to stop. Has normal warmth and cap refill. Also has tenderness over the left tib/fib with normal circulation. Normal exam of upper extremities.
- Axillae (<10 sec): Inspect bilateral axillae
- Neuro (< 20 sec): Test for strength/sensation of each limb and how extremity is held- patient refuses to move bilateral lower extremities but will move toes spontaneously
- Back (<20 sec): Palpation for stepoffs and tenderness along C/T/L spine, rectal inspection

If have errors, will stop and reset, review and restart again.

At end, final checklist of saying yes or no to completion of tasks in primary and secondary survey.

**Case 3**

Title: High Speed Motor Vehicle Accident

Author: Diana Yan

Patient Name: Jacob Smith

Patient Age: 4 year old

Chief Complaint: Motor Vehicle Accident

Objectives:

1. Review the steps of a timely and complete primary and secondary survey.
2. Diagnose and treat right sided pneumothorax during primary survey.
3. Discover findings of bruising over chest on secondary survey.

Expected Run Time: 5-7 min

Simulation Location: Pediatric Trauma Bay

Scenario Participants: Surgical Residents – one is doing primary and secondary survey; one is holding airway; rest are observers; foot of bed/leader is facilitating the simulation

Props needed: C collar on mannequin, gown for mannequin that can be “cut” off, stethoscope, warm blankets off to the side, moulage of bruising across chest (if desired), facemask, tegaderm

Scenario:

Trauma Pager: Level 2 Trauma: 4 yo male MVC. ETA now.

EMS report: This is a 4 year old male who was in a MVC, restrained passenger in back seat. Major damage to front of car with air bag deployment and intrusion. Patient was conscious and crying at scene. No complaints of pain. 135 HR, 85/Palp, 30, 85%O_2_ Sat. Patient was placed on face mask.

Starting vitals: T 36.8, HR 143, RR 32, BP 82/45, Pox 95% on face mask

Mannequin: No breath sounds on right with deviated trachea to the right. No wheezing or crackles with normal lung exam on left, normal bowel sounds, heart sounds (no murmur), eyes open, weakly crying

Resident will start to perform primary survey (No breath sounds on right with deviated trachea to the right):

*if primary survey is interrupted for significant findings or procedures (eg, intubation, chest tube, etc), completion in 10 sec is not required.

- Airway (<10 sec): ask patient to talk and observe that patient is crying and making sounds (airway patency), deviated trachea to the right
- Breathing (<10 sec): Auscultation in two lung fields (no breath sounds on right), have asymmetrical chest rise (minimal on right side) -> Need to state that he would needle decompress and place chest tube at this time and then restart with primary survey
- Circulation (<10 sec): Palpation of central pulses (femoral or brachial) and weak pulses unless did/verbalized needle decompression +/- chest tube, note no obvious bleeding
- Disability (<30 sec): Size and responsiveness of bilateral pupils, GCS 11 (eye spon opening 4, inconsolable 3, withdraws to pain 4)
- Exposure: “cut” clothes off and get warm blankets to prevent hypothermia

If have errors, will stop and reset, review and restart again. Make sure that they have looked at vitals at least once.

Resident will start to perform secondary survey (tenderness over bruising on chest): [Resident will ask for different exam findings and facilitator will give findings. If desired, moulage can indicate abnormal exam findings.]

- Head (<10 sec): Palpation/Inspection of scalp, otoscope exam
- Face (<10 sec): Palpation/Inspection of face, oropharyngeal exam
- Neck (<10 sec): Inspection of anterior neck
- Chest wall (<10 sec): Chest tube in place, no crepitus, tenderness over the chest with bruising from left upper to right lower. All other chest exam findings are normal
- Abdomen (<10 sec): Palpate abdomen for tenderness, soft vs firm, distension
- Pelvis (<10 sec): Palpation for tenderness (including pubic symphysis) and rocked correctly
- Perineum/Urethra (<10 sec): Visual exam of perineum (including meatus in males) and inspect for blood
- Extremities (<20 sec): Visual exam and palpation for tenderness of all non-deformed aspects of 4 limbs
- Axillae (<10 sec): Inspect bilateral axillae
- Neuro (< 20 sec): Test for strength/sensation of each limb and how extremity is held
- Back (<20 sec): Palpation for stepoffs and tenderness along C/T/L spine, rectal inspection

If have errors, will stop and reset, review and restart again.

At end, final checklist of saying yes or no to completion of tasks in primary and secondary survey.

**Case 4**

Title: House Fire

Author: Diana Yan

Patient Name: Jane Rogers

Patient Age: 1 year old

Chief Complaint: Burn

Objectives:

1. Review the steps of a timely and complete primary and secondary survey.
2. Perform a complete skin exam for all burns on body

Expected Run Time: 5-7 min

Simulation Location: Pediatric Trauma Bay

Scenario Participants: Surgical Residents – one is doing primary and secondary survey; one is holding airway; rest are observers; foot of bed/leader is facilitating the simulation

Props needed: No C collar on mannequin, needs a gown for mannequin that can be “cut” off, stethoscope, warm blankets off to the side, moulage of snoot in mouth and deep partial burns on chest wall and abdomen (if desired), tegaderm

Scenario:

Trauma Pager: Level 1 Trauma: 1 yo female in house fire. ETA 10 min.

EMS report: This is a 1 year old female who was found in house fire. Patient will follow commands but sleepy. No complaints of pain. Has burns on body. 148 HR, 125/Palp, 30, 99%O_2_ Sat.

Starting vitals: T 37.9, HR 132, RR 30 BP 110/65, Pox 100% on room air

Mannequin: Normal breath sounds, bowel sounds, heart sounds (no murmur), eyes open, not talking but when asked questions, will whimper.

Resident will start to perform primary survey (whimpering child but will make sounds):

- Airway (<10 sec): ask patient to talk and patient will whimper and make loud “no” sound when touched and then will start to cry (no intubation at this point- airway patency), no tracheal deviation
- Breathing (<10 sec): Auscultation in two lung fields, inspection of equal chest rise
- Circulation (<10 sec): Palpation of pulses, note no obvious bleeding
- Disability (<30 sec): Size and responsiveness of bilateral pupils, GCS 15
- Exposure: “cut” clothes off and get warm blankets to prevent hypothermia

If have errors, will stop and reset, review and restart again. Make sure that they have looked at vitals at least once.

Resident will start to perform secondary survey (soot in mouth, burns on chest, abdomen; will need ETT placement at end – not part of simulation): [Resident will ask for different exam findings and facilitator will give findings. If desired, moulage can indicate abnormal findings.]

- Head (<10 sec): Palpation/Inspection of scalp, otoscope exam
- Face (<10 sec): Palpation/Inspection of face, soot in mouth -> intubate at end of secondary survey given patient is talking (try to keep patient calm)
- Neck (<10 sec): Inspection of anterior neck
- Chest wall (<10 sec): Burns on chest wall that are tender, no crepitus
- Abdomen (<10 sec): Burns on abd and tender, not distended
- Pelvis (<10 sec): Palpation for tenderness (including pubic symphysis) and rocked correctly
- Perineum/Urethra (<10 sec): Visual exam of perineum (including meatus in males) and inspect for blood
- Extremities (<20 sec): Visual exam and palpation for tenderness of all non-deformed aspects of 4 limbs
- Axillae (<10 sec): Inspect bilateral axillae
- Neuro (< 20 sec): Test for strength/sensation of each limb and how extremity is held
- Back (<20 sec): Palpation for stepoffs and tenderness along C/T/L spine, rectal inspection

If have errors, will stop and reset, review and restart again.

At end, final checklist of saying yes or no to completion of tasks in primary and secondary survey.

**Case 5**

Title: Bicycle versus Car

Author: Diana Yan

Patient Name: James Smith

Patient Age: 15 year old

Chief Complaint: Bicycle vs Car

Objective:

1. Review the steps of a timely and complete primary and secondary survey.
2. Recognize a low GCS during primary survey.
3. Discover contusions to head and abrasions on extremities during secondary survey.

Expected Run Time: 5-7 min

Simulation Location: Pediatric Trauma Bay

Scenario Participants: Surgical Residents – one is doing primary and secondary survey; one is holding airway; rest are observers; foot of bed/leader is facilitating the simulation

Props needed: C collar on mannequin, gown for mannequin that can be “cut” off, stethoscope, warm blankets off to the side, Moulage for bump on head and abrasions to arms (if desired)

Scenario:

Trauma Pager: Level 1 Trauma. 15 yo male bicycle vs car. ETA 2 min.

EMS report: This is a 15 year old male who was in bicycle vs car. Patient was riding his bicycle without a helmet when he was hit at approx. 30mph. The patient flew back and hit his head on the concrete. Had +LOC, but in the truck, he woke up and was talking. Vitals stable during transport with HR 90, BP 115/70, RR 18 and 98%O_2_ Sat.

Starting vitals: T 37.1, HR 75, RR 25, BP 130/84, Pox 100% on room air

Mannequin: Normal breath sounds, bowel sounds, heart sounds (no murmur), eyes open, moaning. (Patient is developing increased ICP and becoming unstable.)

Resident will start to perform primary survey (low GCS):

*if primary survey is interrupted for significant findings or procedures (eg, intubation, chest tube, etc), completion in 10 sec is not required.

- Airway (<10 sec): patient is moaning and will make sounds (airway patency), no tracheal deviation
- Breathing (<10 sec): Auscultation in two lung fields, inspection of equal chest rise
- Circulation (<10 sec): Palpation of pulses, note no obvious bleeding
- Disability (<30 sec): Size and responsiveness of bilateral pupils, GCS 8 (eye opening to pain- 2, withdraw flexion to pain- 4; incomprehensible sounds -2) -> need intubation for mental status
- Exposure: “cut” clothes off and get warm blankets to prevent hypothermia

If have errors, will stop and reset, review and restart again. Make sure that they have looked at vitals at least once.

Resident will start to perform secondary survey (intubated patient with contusion to occiput and abrasions to an extremity): [Resident will ask for different exam findings and facilitator will give findings. If desired, moulage can indicate abnormal exam findings.]

- Head (<10 sec): Palpation/Inspection of scalp – contusion to occiput, otoscope exam
- Face (<10 sec): Palpation/Inspection of face
- Neck (<10 sec): Inspection of anterior neck
- Chest wall (<10 sec): Palpation of chest wall, evaluation of crepitus/lacerations
- Abdomen (<10 sec): Palpate abdomen for soft vs firm, distension
- Pelvis (<10 sec): Rock pelvis
- Perineum/Urethra (<10 sec): Visual exam of perineum (including meatus in males) and inspect for blood
- Extremities (<20 sec): Visual exam and palpation for breaks in bone of all non-deformed aspects of 4 limbs; has abrasion to an extremity
- Axillae (<10 sec): Inspect bilateral axillae
- Neuro (< 20 sec): Intubated patient (GCS 3T)
- Back (<20 sec): Palpation for stepoffs along C/T/L spine, rectal inspection

If have errors, will stop and reset, review and restart again.

At end, final checklist of saying yes or no to completion of tasks in primary and secondary survey.

**Case 6**

Title: Shooting

Author: Diana Yan

Patient Name: James Roger

Patient Age: 12 year old

Chief Complaint: Gun Shot Wound

Objectives:

1. Review the steps of a timely and complete primary and secondary survey.
2. Identify need for advance airway, needle decompression/chest tube in primary survey
3. Complete secondary survey and find all GSW wounds

Expected Run Time: 5-7 min

Simulation Location: Pediatric Trauma Bay

Scenario Participants: Surgical Residents – one is doing primary and secondary survey; one is holding airway; rest are observers; foot of bed/leader is facilitating the simulation

Props needed: No C collar on mannequin, gown for mannequin that can be “cut” off, stethoscope, warm blankets off to the side, Moulage of two GSW holes (if desired)

Scenario:

Trauma Pager: Level 1 Trauma: 12 yo male GSW. ETA 4 min.

EMS report: This is a 12 year old male who has one GSW to the right chest. There was significant amount of blood loss at scene. He was talking to use at the scene but has been progressively getting more tired in the ambulance. Started to bag him as we rolled in. Vitals 120 HR, 85/Palp, 99%O_2_ Sat with bagging.

Starting vitals: HR 140, RR 15 (with bagging), BP 79/40, Pox 98%.

Mannequin: Breath sounds on L side only, normal bowel sounds, heart sounds (no murmur), eyes closed, making moaning sounds only

Resident will start to perform primary survey (right hemothorax):

*if primary survey is interrupted for significant findings or procedures (eg, intubation, chest tube, etc), completion in 10 sec is not required.

- Airway (<10 sec): patient is making on moaning sounds, trachea is deviated to left
- Breathing (<10 sec): only breath sounds on left side, not equal chest rise -> needle compression doesn’t get any air, chest tube get blood and then resp exam will normalize (learners should start at top of primary survey after intervention)
- Circulation (<10 sec): weak pulses unless chest tube in place and draining, no obvious active bleeding from GSW
- Disability (<30 sec): Size and responsiveness of bilateral pupils, GCS: 6 (2E- eye open to pain/2V- incomprehensible sounds/2M- abnl extension to pain-decerebrate) – should already be intubated at this point
- Exposure: “cut” clothes off and get warm blankets to prevent hypothermia

If have errors, will stop and reset, review and restart again. Make sure that they have looked at vitals at least once.

Resident will start to perform secondary survey (GSW to right anterior chest with crepitus, chest tube in place, GSW to right upper back, and 2^nd^ GCS is 3T): [Resident will ask for different exam findings and facilitator will give findings. If desired, moulage can indicate abnormal findings.]

- Head (<10 sec): Palpation/Inspection of scalp, otoscope exam
- Face (<10 sec): Palpation/Inspection of face, oropharyngeal exam
- Neck (<10 sec): Inspection of anterior neck (trachea is midline after chest tube placement)
- Chest wall (<10 sec): Crepitus around one right anterior chest GSW, chest tube in place, no rib fractures felt
- Abdomen (<10 sec): Palpate abdomen for soft vs firm, distension
- Pelvis (<10 sec): Rock pelvis
- Perineum/Urethra (<10 sec): Visual exam of perineum (including meatus in males) and inspect for blood
- Extremities (<20 sec): Visual exam and palpation of extremities
- Axillae (<10 sec): Inspect bilateral axillae
- Neuro (< 20 sec): Intubated - GCS 3T
- Back (<20 sec): Palpation for stepoffs along C/T/L spine, has GSW to right upper back (holes + bullets = even number), rectal inspection

If have errors, will stop and reset, review and restart again.

At end, final checklist of saying yes or no to completion of tasks in primary and secondary survey.
